# Rebuttal to Correspondence on “Mortality Pattern of *Poecilus cupreus* Beetles after Repeated Topical Exposure to Insecticide—Stochastic Death or Individual Tolerance?”’

**DOI:** 10.1021/acs.est.4c04127

**Published:** 2024-06-06

**Authors:** Grzegorz Sowa, Agnieszka J. Bednarska, Ryszard Laskowski

**Affiliations:** † Institute of Environmental Sciences, Jagiellonian University, Gronostajowa 7, 30-387 Kraków, Poland; ‡ Institute of Nature Conservation, Polish Academy of Sciences, A. Mickiewicza 33, 31-120 Kraków, Poland

We are pleased that our article^1^ has attracted interest and provoked discussion on what we consider to be an important issue in biology and ecotoxicology: how to correctly interpret mortality in populations exposed to toxic substances (or any stressor). We are also grateful to Roman Ashauer for the interesting and friendly discussion we had before publishing this Correspondence—this is how science and scientists should work. With that said, we must emphasize that neither the aforementioned conversation nor the accompanying Correspondence to which we hereby refer has changed our position on the fundamental error in the assumption that mortality under the influence of toxic substances can be a stochastic phenomenon. We are also convinced that to prove that mortality depends on the individual tolerance of organisms to toxic substances, the data analysis we originally applied using the Kaplan–Meier estimator is completely sufficient. The GUTS model has been added to this work at the request of the reviewers and editor, even though we were (and still are) convinced that it adds nothing to answering our key research question. In addition, the use of the GUTS-SD and GUTS-IT models requires several assumptions, from which the Kaplan–Meier method is free. Via comparison of the mortality curves after three treatments and between oilseed rape and grassland populations, it has been clearly shown that the mortality rate decreased in the grassland population after successive treatments, indicating a gradual elimination of the more susceptible individuals. In contrast, in populations derived from oilseed rape (OSR) fields, and therefore previously exposed to insecticides, mortality rates were similar after the first two doses. This distribution of mortality in these two population types made it possible to show the situations in which mortality may appear to be a stochastic phenomenon, specifically in populations with low individual variability. However, even in the populations from the OSR fields, mortality decreased after the third treatment, indicating also in this case a pattern consistent with the IT model.

However, as the discussion went on to point out possible errors in the application of the GUTS models, we will focus below on addressing the issues raised in the accompanying Correspondence. Before going any further, we must admit that we also spotted a mistake in our data files prepared for GUTS analysis, but luckily, it did not result in any change in the interpretation of the results. When recalculating the original individual lifespan data to mortality rates, as required for GUTS, we had to combine the three sections of the experiment (after each treatment) into one mortality data file, covering the whole experiment. When doing this, we mistakenly added the second and third sections (i.e., after the second and third treatment) on top of the preceding section instead of overlapping the last day of the preceding section with the first day of the following section of the experiment (the last census before the next treatment was on the same day as the treatment). This resulted in an erroneous shift of the second and third treatment days by 1 day; i.e., in GUTS analysis, the first and the second sections were artificially extended by 1 day. However, the mortality pattern was correctly linked with the treatments, so no major change in results should be expected. To check this, we reanalyzed our data, and indeed, the differences in estimated model parameters are negligible or absent (Tables 1 and 2).

**Table 1:** Comparison of the Model Parameters for Meadow Beetles Published by Sowa et al.^1^ and Parameters After Correction of the Data Entry for the One-Day Shift in the Treatment Day^a^

	meadows, original manuscript		meadows, corrected	
model parameters and goodness of fit statistics	SD	IT	SD	IT
*k*_d_ [95% confidence interval (CI)]	3.24 (2.22–4.66)	0.048 (0.026–0.080)	3.33 (2.29–4.77)	0.049 (0.026–0.081)
*m*_w_ (95% CI)	5.42 × 10^–5^ (5.42 × 10^–5^ to 1.14)	2.02 (1.14–3.47)	5.42 × 10^–5^ (5.67 × 10^–5^ to 1.11)	1.96 (1.10–3.35)
*b*_w_ (95% CI)	0.015 (0.011–0.020)	–	0.015 (0.011–0.020)	–
*F*_s_ (95% CI)	–	20 (15.5–20)	–	20 (15.4–20)
NSE	0.683	**0.692**	0.698	**0.719**
NRMSE (%)	34.4	**33.8**	33.5	**32.2**
AIC	**1200.9**	1252.5	**1186.5**	1236.3

a The goodness of fit parameters indicating a better fit (SD vs IT model) are shown in boldface.

**Table 2:** Comparison of the Model Parameters for Oilseed Rape (OSR) Beetles Published by Sowa et al. (1) and Parameters after Correction of the Data Entry for the One-Day Shift in the Treatment Day^a^

	OSR, original manuscript		OSR, corrected	
model parameters and goodness of fit statistics	SD	IT	SD	IT
*k*_d_ (95% CI)	3.19 (1.97–5.59)	0.023 (0.019–0.041)	3.19 (1.97–5.60)	0.024 (0.019–0.042)
*m*_w_ (95% CI)	1.44 (5.42 × 10^–5^ to 4.10)	1.69 (1.16–2.94)	1.44 (5.67 × 10^–5^ to 4.11)	1.74 (1.19–3.01)
*b*_w_ (95% CI)	0.0094 (0.006–0.015)	–	0.0094 (0.006–0.015)	–
*F*_s_ (95% CI)	–	15.97 (6.67–20)	–	15.96 (6.67–20)
NSE	**0.845**	0.807	**0.844**	0.810
NRMSE (%)	**17.1**	19.2	**17.0**	19.1
AIC	**1017.2**	1135.2	**1014.3**	1132.1

a The goodness of fit parameters indicating a better fit (SD vs IT model) are shown in boldface.

Following this correction and clarification, we will move on to respond directly to the specific points raised by Ashauer.

(1) Error in data entry and the “correction” by doubling the treatment day (second treatment, days 29, 29, 29.5, 30, ...; third treatment, days 66, 66, 66.5, 67, ...). This doubling of the treatment day looks more like data manipulation than correction. The lifespan of each individual was recorded for the first 12 h after the treatment to cover the expected high mortality in the first hours and then daily. As GUTS requires mortality rates rather than individual lifespan data, this was recalculated to the population census 12 and 24 h after the treatment and then every 24 h. Hence, the actual data exist for days 27, 28 (here was the second treatment right after the census), 28.5, 29, ..., 63, 64 (the third treatment right after the census), 64.5, 65, ... Therefore, the survival rate was recorded at day 28 and then 12 h after the treatment (28.5) and at day 64 and 12 h after the treatment (64.5). If openGUTS cannot account for that, it is a flaw in the model and not in the data. Ashauer argues that due to that “error” “the exposure implemented in openGUTS for the second and third exposures is 3 times that of the first exposure event”. However, this does not seem to be supported by the graphs generated by openGUTS (Figure 1). Again, if these treatments are erroneously extrapolated in openGUTS, the problem is in the model or software and not in the data. Furthermore, in the experimental design used in our study, with the observed lifespan of each individual, recalculating these into daily mortality rates is a waste of data, but this should not significantly affect the outcome.

**Figure 1:**
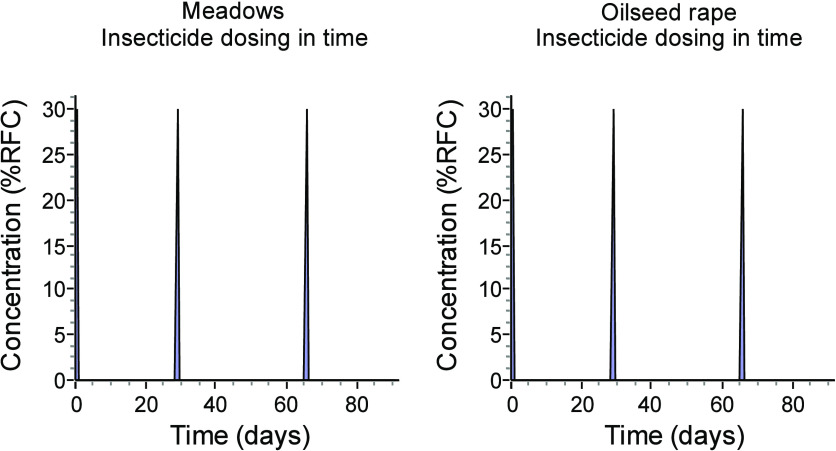
Exposure profiles for the beetles from meadows and oilseed rape fields at the three consecutive doses of the insecticide. Note that all three peaks (exposures) are identical rather than “second and third exposures are 3 times that of the first exposure event” as described by Ashauer.

(2) Ashauer points out that “it is important to also consider the systematically slower damage repair under the IT model”. This is what we see as a major limitation of using GUTS for analyzing experimental data like ours; in contrast to the well-established methods for survival analysis, like the Kaplan–Meier estimator, GUTS has several assumptions implemented in the model that apparently do not always fit the actual data. With experiments based just on survival analysis, the experimenter does not know the damage repair rate that can differ vastly between different groups of animals (for example, it is well-known that toxicants accumulate some animals while others are effective “regulators”; hence, the damage repair rate will be vastly different as the damage occurs in different organs and affects different biochemical pathways). The statement that the damage repair time is “much longer for IT” results from the model assumption and is not the experimentally confirmed fact (by the way, these assumptions seem highly unrealistic because DRT95 values reported by Ashauer suggest that the beetles would need up to several years for damage repair!). Determining the damage and its repair rate requires much more in-depth studies than just observing the mortality rate because evolution and physiological and biochemical constraints can shape these processes differently in different organisms and circumstances.^2,3^ We know that the authors of GUTS claim that the model allows the estimation of toxicokinetics and toxicodynamics from observations of mortality rates alone. We would be happy if this was indeed possible, but unfortunately, this is not the case. To determine toxicokinetics, i.e., the pattern of change in the internal concentration of a toxicant, its concentration needs to be measured at some time intervals. Many studies have shown that toxicants accumulate at different rates even in relatively closely related animals and that the toxicokinetics of the very same toxicant can depend on its concentration in the environment or food and the age of animals (e.g., refs (4−6)). Therefore, the question raised by Ashauer, “How is that explained by selection?”, is irrelevant. We would rather ask the authors of GUTS how they would explain the assumptions about the “dominant rate constant” and the repair rate that are not supported by experimental data. At this point, it is also worth recalling the statement by Ashauer and co-authors from a paper published in 2013:^7^ “Note, however, that the GUTS-SD and GUTS-IT models do not require any a priori assumptions about the speed of compound elimination or the speed of organism recovery. These models let the data speak and capture information on the time course of toxicity in their model parameters during the calibration step”.

(3) The third objection raised by Ashauer is that we wrongly “used the survival data from treatments A-2 and A-3 as control data ... because these organisms had been exposed to the toxicant (all part of treatment P-1) and it cannot be assumed that they have had enough time for complete depuration and damage repair from that first exposure within 4 weeks” so in the “corrected” analysis he “deleted those data from the control survival time series” (SiC!) and estimated the background mortality rate on the basis of only the first acetone treatment (A-1). However, using separate control groups (A-1–A-3) for each consecutive treatment (P-1–P-3), as described in the original article,^1^ was the whole idea behind the experiment! These control groups were created precisely to account for possible delayed effects caused by incomplete depuration or repair of damage. Removing these data from the analysis is a clear manipulation and is against the experimental design and the hypothesis tested. The only comment among those mentioned in this section of the accompanying Correspondence with which we can agree is that the GUTS analysis could probably be improved using mortality observations recorded 2, 4, 6, 8, 10, and 12 h after exposure (and this is why we mentioned above that recalculating the precisely recorded lifetimes, as used in the Kaplan–Meier analysis, is a waste of data; even recording the mortality after 2, 4, 6, 8, ... h would be less precise than using the actual lifespans). See also the statement cited above from ref (7).

(4) The final remark made by Ashauer that we did not consider differences in body mass between individuals and that these differences alone could be responsible for the variable sensitivity of the beetles is sensible. Unfortunately, in the case of carabids fed *ad libitum* during the experiment, the usefulness of body mass would also be questionable because these beetles can consume huge amounts of food and their body mass can fluctuate greatly,^8^ with only little or no relationship to protein and fat contents, which could theoretically be responsible for the variation in their susceptibility to toxicants. Hence, our study was based on the comparison between meadow and OSR populations which did not differ in body mass as reported in the original article.^1^ In this context, it is worth noting that OSR beetles revealed the IT-like mortality pattern when comparing the effects after the second and third doses even if the first two doses resulted in similar mortality rates (the SD-like pattern). If the variance in body mass was responsible for that difference, we would obtain the IT-like pattern in all cases. In addition, in several articles using GUTS analysis, Ashauer and co-authors do not even mention the body mass of the studied animals or conduct experiments on groups of individuals, making it impossible to consider individual body mass (cf. refs (7) and (9)). We must therefore consider this remark to be somewhat inconsistent, given the history of the research carried out using GUTS.

Finally, some estimates of the “corrected” version of openGUTS analysis of our data by Ashauer are nonsense, proving that the modeling is highly incorrect. Specifically, the LC50 for the OSR population on the first day after the first insecticide application estimated by Ashauer is 9.341% recommended application concentration (RAC). However, actual mortality after 24 h was 28.1% at 30% RAC, meaning that LC50 must be substantially higher than that. Indeed, according to our estimates, LC50 is 107.6% RAC and LC20 36.02 RAC, values that are much more sensible than those reported by Ashauer. The errors in the “corrected” model are also clearly visible from a comparison of the LC50, LC20, and LC10 values estimated by Ashauer. For example, on the first day after insecticide application, these three values barely differ from one another and the differences are statistically nonsignificant, as one can see from 95% confidence intervals: LC50 = 9.341 (8.237–67.78), LC20 = 8.63 (8.018–21.88), and LC10 = 8.355 (5.65–11). These results are, of course, impossible, as they would mean that at around 8.4–9.3% RAC, the mortality rate could just as easily be around 10% as 50% (while in fact it was 28.1% at 30% RAC). Our estimates are much more reasonable and differ significantly from one another: LC50 = 107.6 (73.52–159.6), LC20 = 36.02 (26.34–52.12), and LC10 = 18.07 (13.41–25.37).

In conclusion, we remain of the opinion that the GUTS analysis was not needed in our study to test the IT versus SD hypothesis and indeed did not contribute anything new compared to the Kaplan–Meier analysis. Hence, whatever happened with the GUTS analysis, it does not undermine our conclusion. However, the “correction” by Ashauer seems very incorrect (removing a major part of the data for control treatments, doubling selected days of the experiment) and resulted in nonsense estimates. Although it was not our intention to focus on criticizing GUTS, the need to address the accompanying Correspondence by Ashauer forced us to examine it more closely. The completely flawed estimates of LD50s (etc.) in the “corrected” analysis, the lack of a good fit to the data, and the claim that control data for the separate sections of the experiment cannot be used brought us to the conclusion that there are serious problems with the openGUTS model that need to be urgently addressed if it is to be used in ecological risk assessment and ecotoxicology in general.

With regard to the substance, the fact that “The concepts of SD and IT have a long history, going back a century” cannot be used as a proof of concept. The theory of natural selection has a longer history, and despite many efforts, no one has succeeded in disproving it. We feel somewhat embarrassed that we are engaged in proving something that had been proven by Charles Darwin more than 150 years ago and encapsulated by Herbert Spencer in the famous phrase “survival of the fittest”. Accepting the idea that mortality in populations is a stochastic process would mean that we instead accept the “survival of the luckiest” theory. As biologists, we cannot agree with this (although we do not deny that a little luck comes in handy). From a practical point of view, we recommend removing the SD model from GUTS and focusing on polishing the IT part.

## Supporting Information Available

Insecticide dosing times for beetles in meadows and oilseed rape fields (PDF)
openGUTS report for beetles in meadows (PDF)
openGUTS report for beetles in oilseed rape fields (PDF)
