# Supplementary figures and images for: Rebuttal to Correspondence on “Mortality Pattern of Poecilus cupreus Beetles after Repeated Topical Exposure to Insecticide—Stochastic Death or Individual Tolerance?”’
Source: Environ Sci Technol. 2024 Jun 6;58(24):10877–80. doi: 10.1021/acs.est.4c04127 (PMC11191582; doi:10.1021/acs.est.4c04127)

Meadows  
Insecticide dosing in time

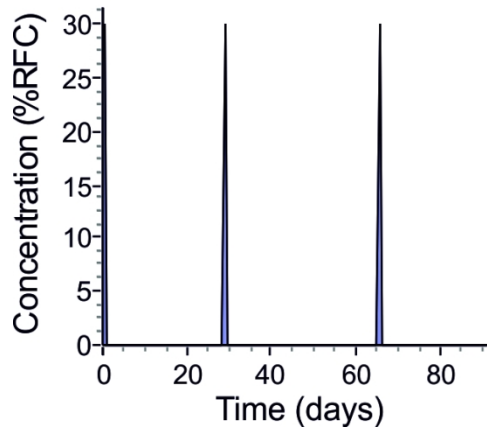

Oilseed rape  
Insecticide dosing in time

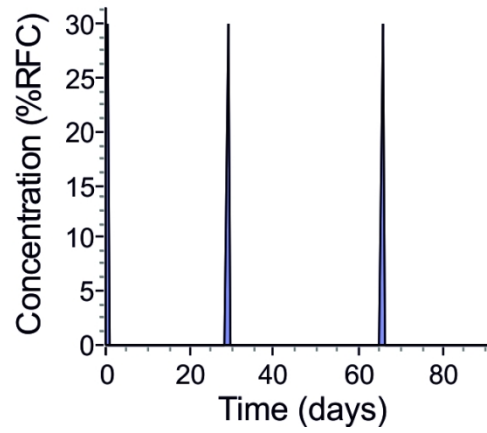

112x59mm (300 x 300 DPI)

Supplement: Supplementary file 1 — es4c04127_si_001.pdf [file es4c04127_si_001.pdf]
