# Supplementary material for: Rebuttal to Correspondence on “Mortality Pattern of Poecilus cupreus Beetles after Repeated Topical Exposure to Insecticide—Stochastic Death or Individual Tolerance?”’
Source: Environ Sci Technol. 2024 Jun 6;58(24):10877–80. doi: 10.1021/acs.est.4c04127 (PMC11191582; doi:10.1021/acs.est.4c04127)
Supplement: Supplementary file 2 — es4c04127_si_002.pdf [file es4c04127_si_002.pdf]

openGUTS Report

Project:

GUTS report M - days 0-28-64

Project file:

No project file saved or loaded

Project description (optional):

Meadows: Data recalculated to correct for the number of beetles moved to acetone control after the 2nd and 3rd dosing to enable GUTS estimation throughout the whole experiment.

Software version:

openGUTS - 1.1

Date of report creation:

28/03/2024 18:32:44

Calibration

Calibration input data

Data set 1

File: GUTS_M

Description (optional):

Meadows: Data recalculated to correct for the number of beetles moved to acetone control after the 2nd and 3rd dosing to enable GUTS estimation throughout the whole experiment.

Control group: 'M-A-corrrected'

Survival data of input data set 1:

Time [d]	M-A-corrrected	M-P-corrected
0	40	160
0.5	40	131
1	39	110
2	39	110
3	39	110
4	39	107
5	39	107
6	38	104
7	38	104
8	38	102
9	36	101
10	36	97
11	36	94
12	35	93
13	35	93
14	35	88
15	35	88
16	35	87
17	34	86
18	34	85
19	34	85
20	34	83

21	34	81
22	34	80
23	34	77
24	33	77
25	33	77
26	33	77
27	33	76
28	33	74
28.5	33	68
29	33	68
30	33	66
31	33	64
32	33	64
33	32	64
34	32	64
35	32	64
36	32	64
37	32	64
38	32	64
39	31	64
40	31	64
41	30	62
42	30	60
43	30	60
44	30	60
45	30	60
46	30	60
47	30	60
48	30	60
49	30	60
50	30	60
51	30	60
52	30	60
53	30	60

54	30	60
55	30	60
56	30	60
57	30	60
58	30	60
59	29	60
60	29	60
61	29	60
62	29	60
63	29	60
64	29	60
64.5	29	56
65	29	56
66	29	56
67	29	56
68	29	56
69	29	56
70	29	56
71	29	56
72	21	56
73	21	56
74	21	56
75	21	56
76	21	56
77	21	56
78	21	56
79	21	56
80	21	56
81	21	56
82	21	56
83	21	56
84	21	56
85	21	56
86	21	56

87	21	56
88	21	56
89	21	56

Concentration data of input data set 1:

Time [d]	M-A-corrrected	M-P-corrected
0	0	30
0.5	0	0
1	0	0
2	0	0
3	0	0
4	0	0
5	0	0
6	0	0
7	0	0
8	0	0
9	0	0
10	0	0
11	0	0
12	0	0
13	0	0
14	0	0
15	0	0
16	0	0
17	0	0
18	0	0
19	0	0
20	0	0
21	0	0
22	0	0
23	0	0
24	0	0
25	0	0
26	0	0

27	0	0
28	0	30
28.5	0	0
29	0	0
30	0	0
31	0	0
32	0	0
33	0	0
34	0	0
35	0	0
36	0	0
37	0	0
38	0	0
39	0	0
40	0	0
41	0	0
42	0	0
43	0	0
44	0	0
45	0	0
46	0	0
47	0	0
48	0	0
49	0	0
50	0	0
51	0	0
52	0	0
53	0	0
54	0	0
55	0	0
56	0	0
57	0	0
58	0	0
59	0	0

60	0	0
61	0	0
62	0	0
63	0	0
64	0	30
64.5	0	0
65	0	0
66	0	0
67	0	0
68	0	0
69	0	0
70	0	0
71	0	0
72	0	0
73	0	0
74	0	0
75	0	0
76	0	0
77	0	0
78	0	0
79	0	0
80	0	0
81	0	0
82	0	0
83	0	0
84	0	0
85	0	0
86	0	0
87	0	0
88	0	0
89	0	0

Calibration settings

Calibration parameter settings for GUTS-RED-SD:

Parameter	Fit	Min	Max	Scale
kd	Yes	0.0005763	143.8	Log
mw	Yes	5.666E-5	29.7	Norm
hb	No	0.007074	0.007074	Norm
bw	Yes	3.946E-5	32471	Log
Fs	No	1	1	Norm

Calibration parameter settings for GUTS-RED-IT:

Parameter	Fit	Min	Max	Scale
kd	Yes	0.0005763	32.91	Log
mw	Yes	5.666E-5	60	Log
hb	No	0.007074	0.007074	Norm
bw	No	Inf	Inf	Norm
Fs	Yes	1.05	20	Log

Note:

Background hazard (hb) was prefitted to control.

Calibration results

Fitted parameters for GUTS-RED-SD:

Best fit parameter values and their 95% CI

kd: 3.325 (2.292 - 4.766)

mw: 5.666E-5 (5.666E-5* - 1.109)

bw: 0.01509 (0.01142 - 0.02016)

* edge of 95% parameter CI has run into a boundary

(this may also affect CIs of other parameters)

Goodness of fit for calibration data (GUTS-RED-SD):

Model efficiency (NSE, r-square): 0.6979

Normalised root-means-square error (NRMSE): 33.52 %

Minus log-likelihood (MLL): 590.27

AIC: 1186.54

Survival probability prediction error (SPPE) for each treatment:

Data set	Treatment	Value
1	M-A-corrrected	-0.78 %
1	M-P-corrected	10.87 %

GUTS-RED-SD results table for LCx,t [%RFD], with 95% CI:

Time [d]	LC50	LC20	LC10
1	64.7 (48.86 - 88.2)	20.83 (15.77 - 28.39)	9.835 (7.444 - 13.41)
2	27.03 (20.71 - 36.22)	8.702 (6.679 - 11.66)	4.109 (3.154 - 5.544)
3	17.02 (13.1 - 22.74)	5.479 (4.217 - 7.32)	2.587 (1.991 - 3.72)
4	12.42 (9.571 - 16.57)	3.998 (3.081 - 5.354)	1.888 (1.455 - 2.965)
7	6.858 (5.294 - 9.133)	2.208 (1.704 - 3.236)	1.042 (0.8047 - 2.111)
14	3.354 (2.591 - 4.514)	1.08 (0.8342 - 2.121)	0.5098 (0.3939 - 1.597)
21	2.219 (1.716 - 3.218)	0.7145 (0.5523 - 1.774)	0.3374 (0.2608 - 1.443)
28	1.659 (1.282 - 2.659)	0.534 (0.4128 - 1.609)	0.2522 (0.1949 - 1.368)
42	1.102 (0.8518 - 2.126)	0.3547 (0.2743 - 1.454)	0.1675 (0.1295 - 1.293)
50	0.9244 (0.7147 - 1.961)	0.2976 (0.2301 - 1.404)	0.1406 (0.1087 - 1.27)
100	0.4608 (0.3563 - 1.541)	0.1484 (0.1147 - 1.275)	0.0701 (0.0542 - 1.209)

Plots for GUTS-RED-SD calibration:

Parameter space plot for the calibration of GUTS-RED-SD:

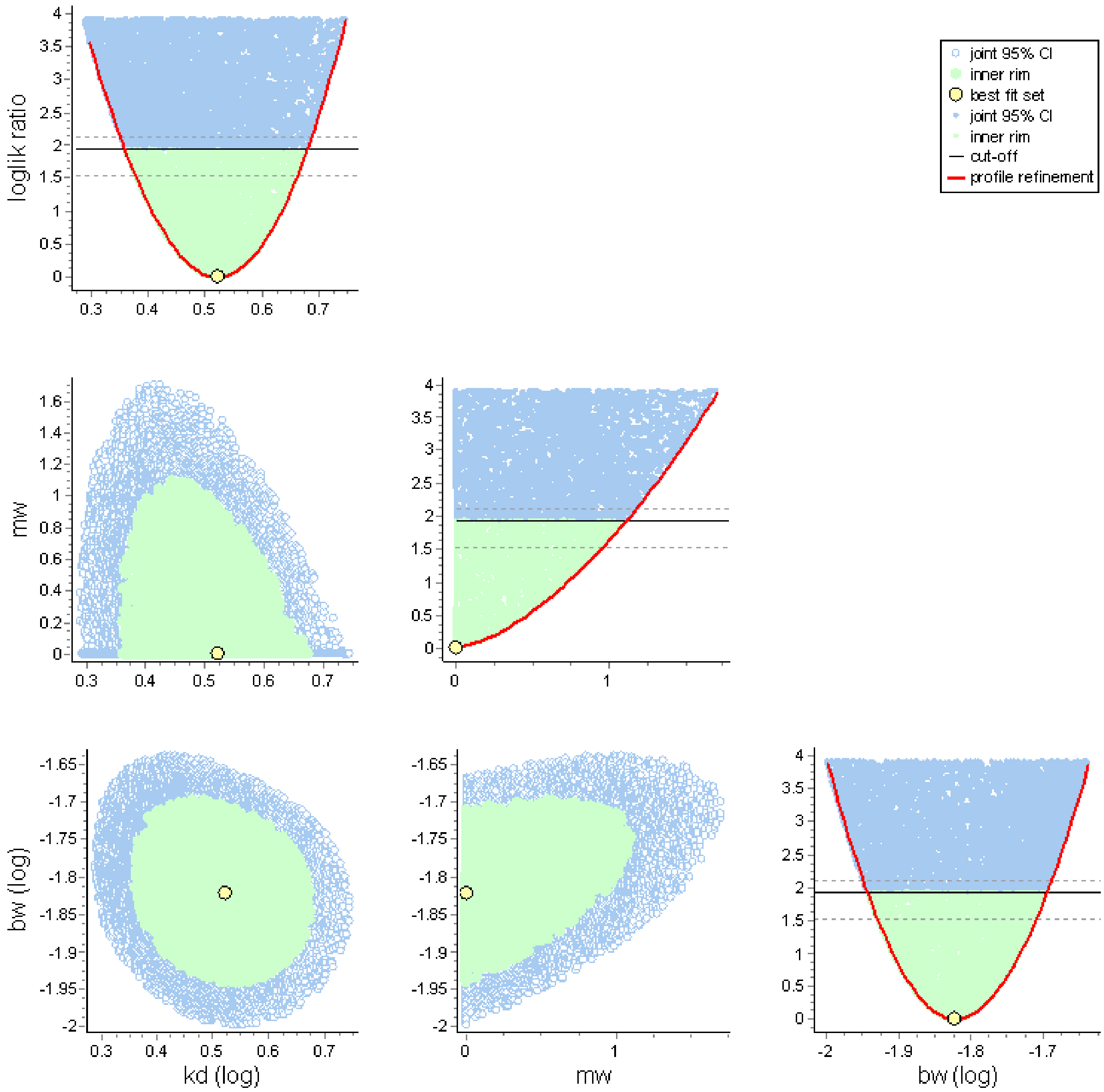

Exposure, damage and survival plots for the calibration of GUTS-RED-SD:

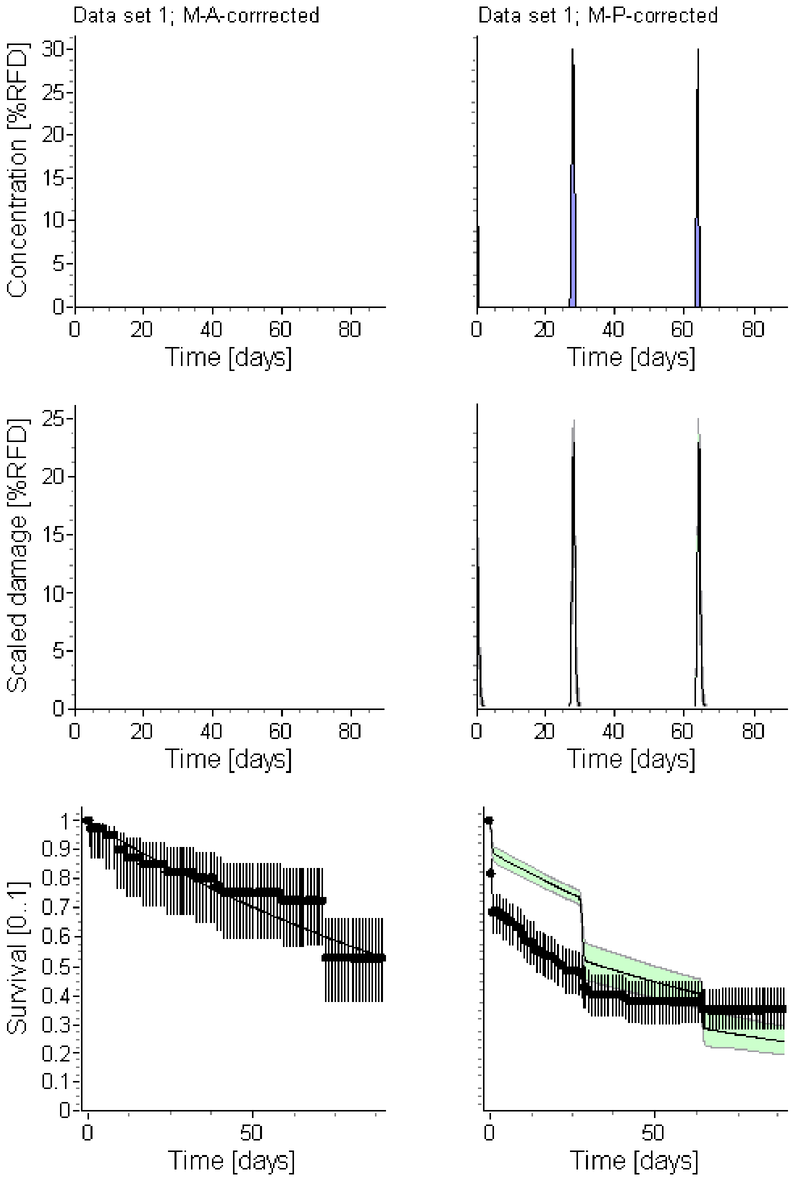

Observed vs. Predicted survival plot for the calibration of GUTS-RED-SD:

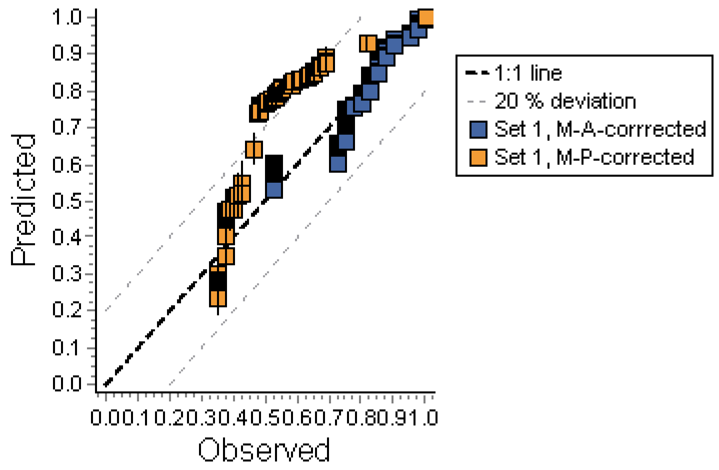

Observed vs. Predicted deaths plot for the calibration of GUTS-RED-SD:

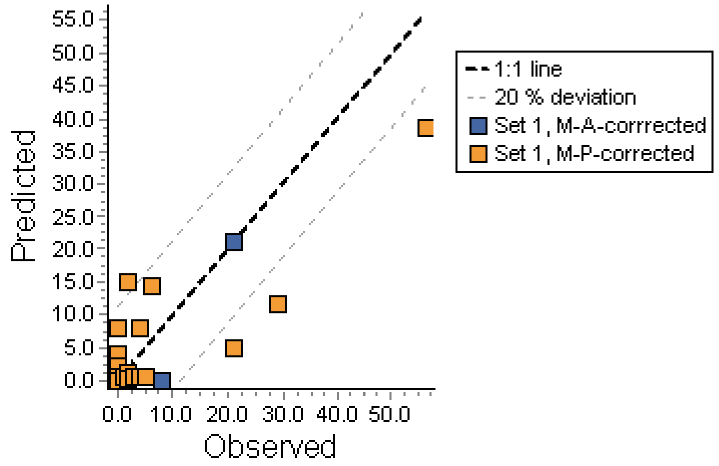

LCx versus time with confidence intervals (plotted for 16 days, GUTS-RED-SD):

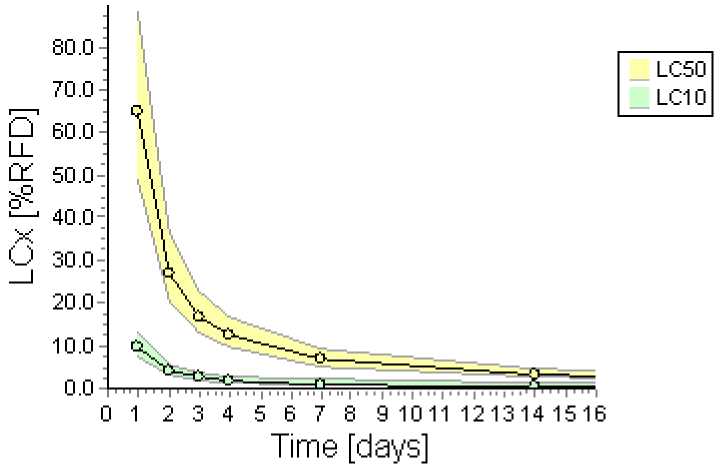

Fitted parameters for GUTS-RED-IT:

Best fit parameter values and their 95% CI

kd: 0.04856 (0.02586 - 0.0807)

mw: 1.961 (1.104 - 3.346)

Fs: 20 (15.39 - 20*)

* edge of 95% parameter CI has run into a boundary

(this may also affect CIs of other parameters)

Goodness of fit for calibration data (GUTS-RED-IT):

Model efficiency (NSE, r-square): 0.7188

Normalised root-means-square error (NRMSE): 32.21 %

Minus log-likelihood (MLL): 615.13

AIC: 1236.26

Survival probability prediction error (SPPE) for each treatment:

Data set	Treatment	Value
1	M-A-corrrected	-0.78 %
1	M-P-corrected	1.306 %

GUTS-RED-IT results table for LCx,t [%RFD], with 95% CI:

Time [d]	LC50	LC20	LC10
1	41.38 (29.99 - 58.73)	13.32 (9.657 - 18.9)	6.862 (4.977 - 9.74)
2	21.19 (15.4 - 29.94)	6.821 (4.96 - 9.637)	3.515 (2.556 - 4.966)
3	14.47 (10.55 - 20.36)	4.657 (3.397 - 6.553)	2.399 (1.751 - 3.377)
4	11.11 (8.123 - 15.6)	3.576 (2.616 - 5.021)	1.843 (1.348 - 2.587)
7	6.806 (5.002 - 9.49)	2.191 (1.61 - 3.055)	1.129 (0.8295 - 1.574)
14	3.976 (2.892 - 5.596)	1.28 (0.9308 - 1.801)	0.6594 (0.4796 - 0.928)
21	3.068 (2.204 - 4.422)	0.9875 (0.7096 - 1.423)	0.5088 (0.3656 - 0.7334)
28	2.639 (1.869 - 3.939)	0.8494 (0.6027 - 1.268)	0.4377 (0.3105 - 0.6532)
42	2.255 (1.513 - 3.547)	0.7258 (0.487 - 1.142)	0.3739 (0.251 - 0.5882)
50	2.151 (1.404 - 3.47)	0.6924 (0.452 - 1.117)	0.3568 (0.2329 - 0.5756)
100	1.977 (1.151 - 3.384)	0.6363 (0.3704 - 1.089)	0.3279 (0.1908 - 0.5612)

Plots for GUTS-RED-IT calibration:

Parameter space plot for the calibration of GUTS-RED-IT:

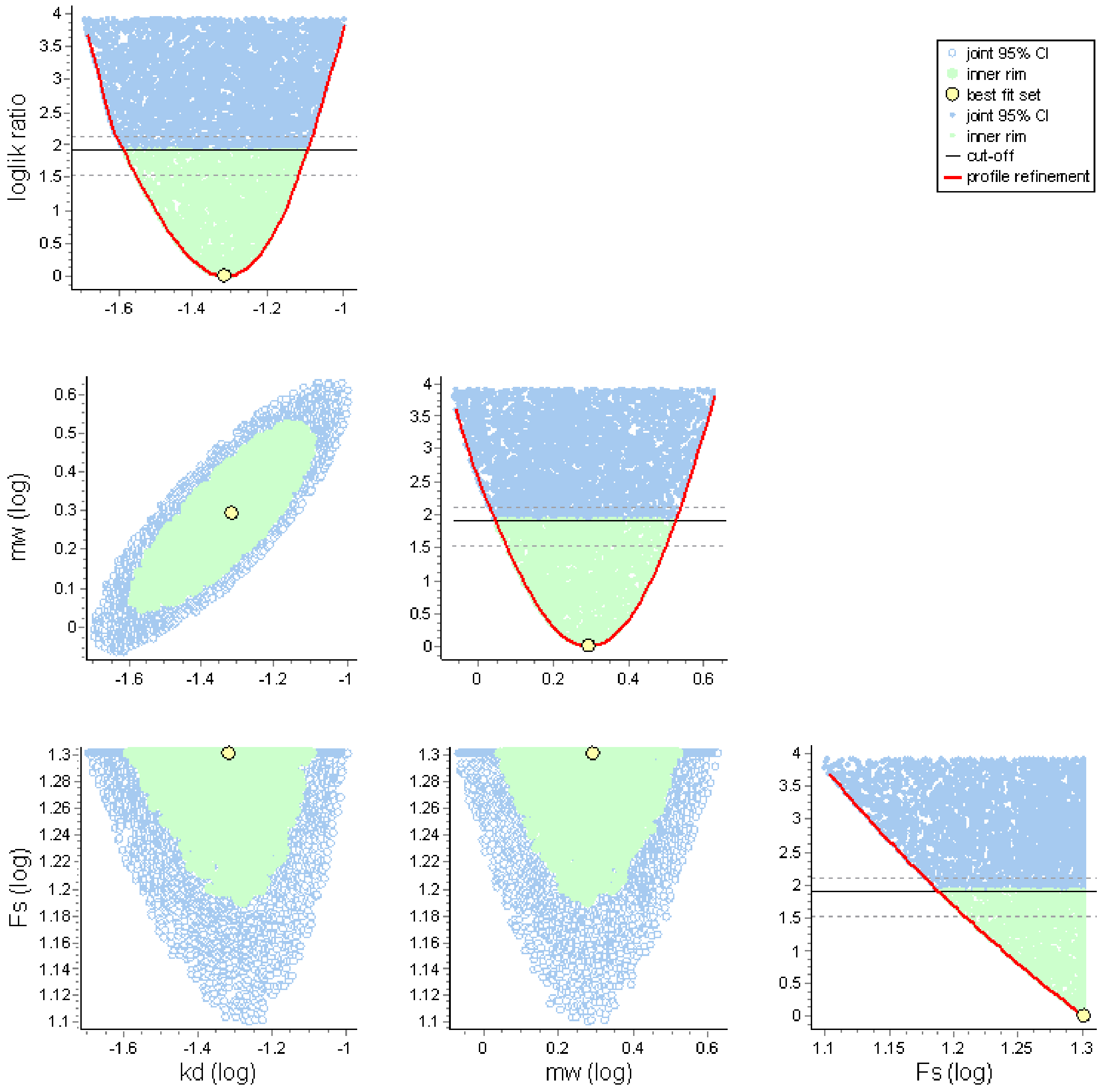

Exposure, damage and survival plots for the calibration of GUTS-RED-IT:

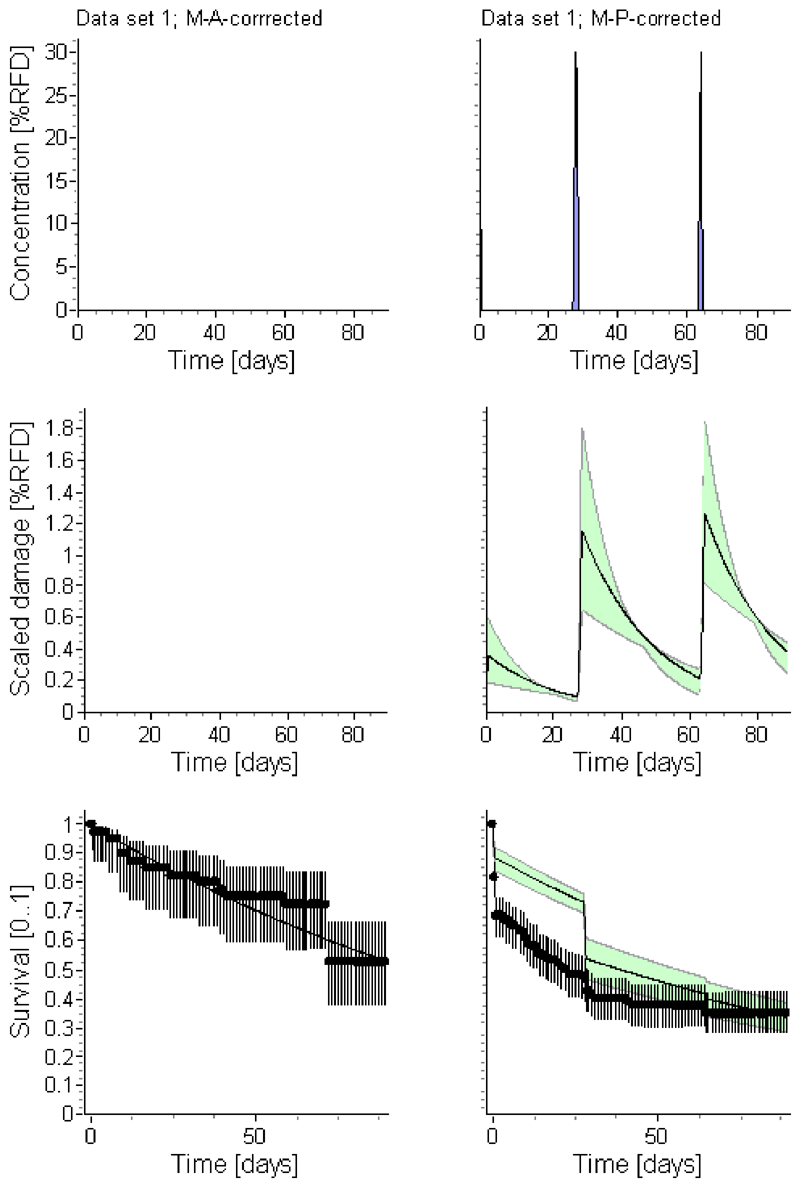

Observed vs. Predicted survival plot for the calibration of GUTS-RED-IT:

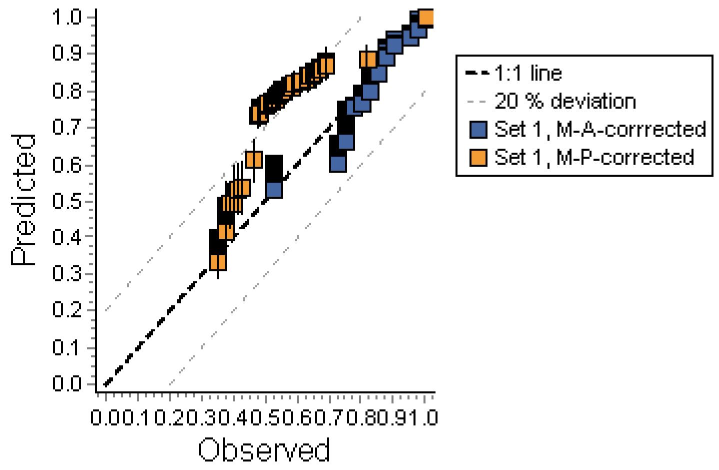

Observed vs. Predicted deaths plot for the calibration of GUTS-RED-IT:

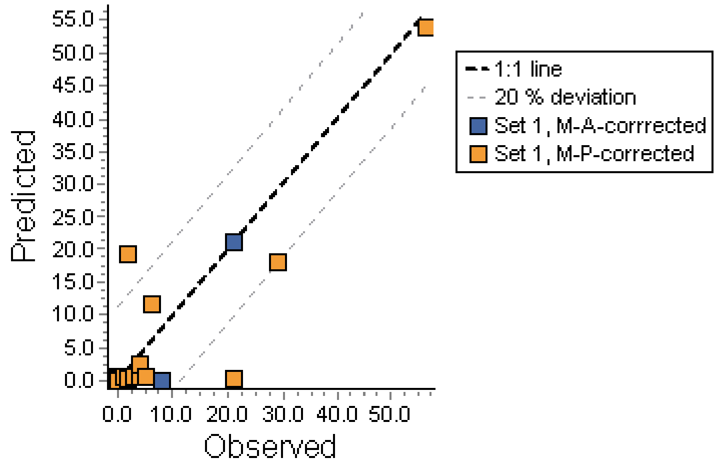

LCx versus time with confidence intervals (plotted for 16 days, GUTS-RED-IT):

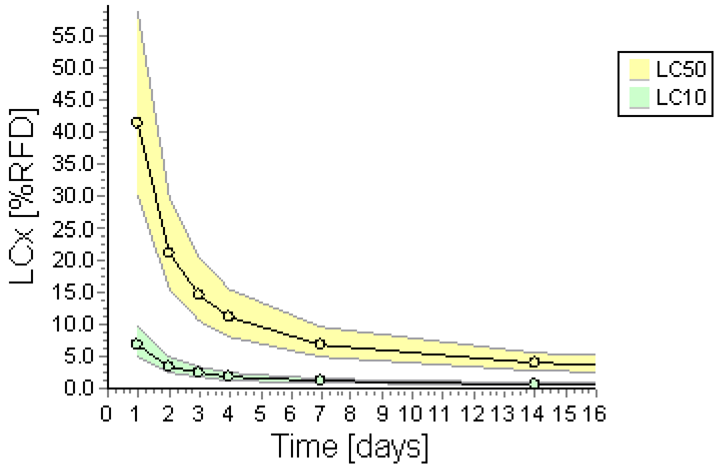

Validation

No validation performed!

Predictions

No predictions performed!
