# Supplementary material for: Rebuttal to Correspondence on “Mortality Pattern of Poecilus cupreus Beetles after Repeated Topical Exposure to Insecticide—Stochastic Death or Individual Tolerance?”’
Source: Environ Sci Technol. 2024 Jun 6;58(24):10877–80. doi: 10.1021/acs.est.4c04127 (PMC11191582; doi:10.1021/acs.est.4c04127)
Supplement: Supplementary file 3 — es4c04127_si_003.pdf [file es4c04127_si_003.pdf]

# openGUTS Report

**Project:**

GUTS report OSR - 20240327

**Project file:**

No project file saved or loaded

**Project description (optional):**

OSR: Data recalculated to correct for the number of beetles moved to acetone control after the 2nd and 3rd dosing to enable GUTS estimation throughout the whole experiment.

**Software version:**

openGUTS - 1.1

**Date of report creation:**

27/03/2024 19:15:16

# Calibration

## Calibration input data

**Data set 1**

File: GUTS_OSR

Description (optional):

OSR: Data recalculated to correct for the number of beetles moved to acetone control after the 2nd and 3rd dosing to enable GUTS estimation throughout the whole experiment.

Control group: 'OSR-A-corrrected'

### Survival data of input data set 1:

| Time [d] | OSR-A-corrrected | OSR-P-corrected |
|---|---|---|
| 0 | 40 | 160 |
| 0.5 | 40 | 148 |
| 1 | 40 | 140 |
| 2 | 40 | 140 |
| 3 | 40 | 138 |
| 4 | 40 | 138 |
| 5 | 40 | 138 |
| 6 | 40 | 136 |
| 7 | 40 | 136 |
| 8 | 40 | 134 |
| 9 | 40 | 134 |
| 10 | 40 | 133 |
| 11 | 38 | 132 |
| 12 | 37 | 131 |
| 13 | 37 | 131 |
| 14 | 37 | 131 |
| 15 | 37 | 131 |
| 16 | 37 | 130 |
| 17 | 37 | 130 |
| 18 | 37 | 127 |
| 19 | 37 | 124 |
| 20 | 37 | 121 |

| | | |
|---|---|---|
| 21 | 36 | 121 |
| 22 | 36 | 120 |
| 23 | 36 | 117 |
| 24 | 36 | 117 |
| 25 | 36 | 117 |
| 26 | 35 | 117 |
| 27 | 35 | 115 |
| 28 | 35 | 115 |
| 28.5 | 35 | 101 |
| 29 | 34 | 101 |
| 30 | 34 | 101 |
| 31 | 34 | 101 |
| 32 | 34 | 101 |
| 33 | 34 | 97 |
| 34 | 33 | 93 |
| 35 | 33 | 91 |
| 36 | 33 | 89 |
| 37 | 33 | 89 |
| 38 | 33 | 89 |
| 39 | 33 | 85 |
| 40 | 32 | 85 |
| 41 | 32 | 85 |
| 42 | 32 | 85 |
| 43 | 31 | 85 |
| 44 | 31 | 83 |
| 45 | 31 | 83 |
| 46 | 31 | 83 |
| 47 | 31 | 81 |
| 48 | 31 | 79 |
| 49 | 31 | 79 |
| 50 | 31 | 79 |
| 51 | 30 | 77 |
| 52 | 30 | 77 |
| 53 | 30 | 77 |

| | | |
|---|---|---|
| 54 | 30 | 77 |
| 55 | 30 | 77 |
| 56 | 30 | 77 |
| 57 | 30 | 77 |
| 58 | 30 | 77 |
| 59 | 30 | 77 |
| 60 | 30 | 77 |
| 61 | 30 | 77 |
| 62 | 30 | 77 |
| 63 | 29 | 77 |
| 64 | 29 | 77 |
| 64.5 | 29 | 68 |
| 65 | 29 | 68 |
| 66 | 29 | 68 |
| 67 | 29 | 68 |
| 68 | 29 | 68 |
| 69 | 29 | 68 |
| 70 | 29 | 68 |
| 71 | 29 | 68 |
| 72 | 29 | 68 |
| 73 | 27 | 68 |
| 74 | 27 | 68 |
| 75 | 27 | 68 |
| 76 | 27 | 68 |
| 77 | 27 | 68 |
| 78 | 27 | 68 |
| 79 | 27 | 68 |
| 80 | 27 | 68 |
| 81 | 27 | 68 |
| 82 | 27 | 68 |
| 83 | 27 | 68 |
| 84 | 27 | 68 |
| 85 | 27 | 68 |
| 86 | 27 | 68 |

| | | |
|---|---|---|
| 87 | 27 | 68 |
| 88 | 27 | 68 |
| 89 | 27 | 68 |

## Concentration data of input data set 1:

| Time [d] | OSR-A-corrrected | OSR-P-corrected |
|---|---|---|
| 0 | 0 | 30 |
| 0.5 | 0 | 0 |
| 1 | 0 | 0 |
| 2 | 0 | 0 |
| 3 | 0 | 0 |
| 4 | 0 | 0 |
| 5 | 0 | 0 |
| 6 | 0 | 0 |
| 7 | 0 | 0 |
| 8 | 0 | 0 |
| 9 | 0 | 0 |
| 10 | 0 | 0 |
| 11 | 0 | 0 |
| 12 | 0 | 0 |
| 13 | 0 | 0 |
| 14 | 0 | 0 |
| 15 | 0 | 0 |
| 16 | 0 | 0 |
| 17 | 0 | 0 |
| 18 | 0 | 0 |
| 19 | 0 | 0 |
| 20 | 0 | 0 |
| 21 | 0 | 0 |
| 22 | 0 | 0 |
| 23 | 0 | 0 |
| 24 | 0 | 0 |
| 25 | 0 | 0 |
| 26 | 0 | 0 |

| | | |
|---|---|---|
| 27 | 0 | 0 |
| 28 | 0 | 30 |
| 28.5 | 0 | 0 |
| 29 | 0 | 0 |
| 30 | 0 | 0 |
| 31 | 0 | 0 |
| 32 | 0 | 0 |
| 33 | 0 | 0 |
| 34 | 0 | 0 |
| 35 | 0 | 0 |
| 36 | 0 | 0 |
| 37 | 0 | 0 |
| 38 | 0 | 0 |
| 39 | 0 | 0 |
| 40 | 0 | 0 |
| 41 | 0 | 0 |
| 42 | 0 | 0 |
| 43 | 0 | 0 |
| 44 | 0 | 0 |
| 45 | 0 | 0 |
| 46 | 0 | 0 |
| 47 | 0 | 0 |
| 48 | 0 | 0 |
| 49 | 0 | 0 |
| 50 | 0 | 0 |
| 51 | 0 | 0 |
| 52 | 0 | 0 |
| 53 | 0 | 0 |
| 54 | 0 | 0 |
| 55 | 0 | 0 |
| 56 | 0 | 0 |
| 57 | 0 | 0 |
| 58 | 0 | 0 |
| 59 | 0 | 0 |

| | | |
|---|---|---|
| 60 | 0 | 0 |
| 61 | 0 | 0 |
| 62 | 0 | 0 |
| 63 | 0 | 0 |
| 64 | 0 | 30 |
| 64.5 | 0 | 0 |
| 65 | 0 | 0 |
| 66 | 0 | 0 |
| 67 | 0 | 0 |
| 68 | 0 | 0 |
| 69 | 0 | 0 |
| 70 | 0 | 0 |
| 71 | 0 | 0 |
| 72 | 0 | 0 |
| 73 | 0 | 0 |
| 74 | 0 | 0 |
| 75 | 0 | 0 |
| 76 | 0 | 0 |
| 77 | 0 | 0 |
| 78 | 0 | 0 |
| 79 | 0 | 0 |
| 80 | 0 | 0 |
| 81 | 0 | 0 |
| 82 | 0 | 0 |
| 83 | 0 | 0 |
| 84 | 0 | 0 |
| 85 | 0 | 0 |
| 86 | 0 | 0 |
| 87 | 0 | 0 |
| 88 | 0 | 0 |
| 89 | 0 | 0 |

## Calibration settings

Calibration parameter settings for GUTS-RED-SD:

| Parameter | Fit | Min | Max | Scale |
|---|---|---|---|---|
| kd | Yes | 0.0005763 | 143.8 | Log |
| mw | Yes | 5.666E-5 | 29.7 | Norm |
| hb | No | 0.004508 | 0.004508 | Norm |
| bw | Yes | 3.946E-5 | 32471 | Log |
| Fs | No | 1 | 1 | Norm |

Calibration parameter settings for GUTS-RED-IT:

| Parameter | Fit | Min | Max | Scale |
|---|---|---|---|---|
| kd | Yes | 0.0005763 | 143.8 | Log |
| mw | Yes | 5.666E-5 | 60 | Norm |
| hb | No | 0.004508 | 0.004508 | Norm |
| bw | No | Inf | Inf | Norm |
| Fs | Yes | 1.05 | 20 | Log |

Note:

Background hazard (hb) was prefitted to control.

## Calibration results

**Fitted parameters for GUTS-RED-SD:**

Best fit parameter values and their 95% CI

kd: 3.191 (1.965 - 5.596)

mw: 1.435 (5.666E-5* - 4.112)

bw: 0.009388 (0.006055 - 0.01492)

* edge of 95% parameter CI has run into a boundary

(this may also affect CIs of other parameters)

**Goodness of fit for calibration data (GUTS-RED-SD):**

Model efficiency (NSE, r-square): 0.8484

Normalised root-means-square error (NRMSE): 16.95 %

Minus log-likelihood (MLL): 554.14

AIC: 1114.27

Survival probability prediction error (SPPE) for each treatment:

| Data set | Treatment | Value |
|---|---|---|
| 1 | OSR-A-corrrected | 0.5492 % |
| 1 | OSR-P-corrected | -1.93 % |

**GUTS-RED-SD results table for LCx,t [[C]], with 95% CI:**

| Time [d] | LC50 | LC20 | LC10 |
|---|---|---|---|
| 1 | 107.6 (73.52 - 159.6) | 36.02 (26.34 - 52.12) | 18.07 (13.41 - 25.37) |
| 2 | 45.46 (32.07 - 67.35) | 15.78 (12.07 - 22.12) | 8.329 (6.098 - 11.16) |
| 3 | 29.08 (21 - 42.86) | 10.44 (7.944 - 14.22) | 5.757 (3.921 - 7.917) |
| 4 | 21.58 (15.91 - 31.47) | 7.993 (5.967 - 10.69) | 4.58 (2.887 - 6.764) |
| 7 | 12.54 (9.591 - 17.66) | 5.047 (3.393 - 7.123) | 3.163 (1.604 - 5.549) |
| 14 | 6.859 (5.103 - 9.141) | 3.196 (1.668 - 5.538) | 2.274 (0.7875 - 4.808) |
| 21 | 5.023 (3.426 - 7.062) | 2.599 (1.105 - 5.056) | 1.989 (0.5219 - 4.575) |
| 28 | 4.115 (2.567 - 6.238) | 2.303 (0.8264 - 4.82) | 1.848 (0.3902 - 4.467) |
| 42 | 3.214 (1.706 - 5.528) | 2.011 (0.5493 - 4.588) | 1.708 (0.2594 - 4.363) |
| 50 | 2.928 (1.432 - 5.303) | 1.918 (0.461 - 4.515) | 1.664 (0.2177 - 4.331) |
| 100 | 2.179 (0.7141 - 4.715) | 1.675 (0.2299 - 4.338) | 1.549 (0.1086 - 4.246) |

**Plots for GUTS-RED-SD calibration:**

## Parameter space plot for the calibration of GUTS-RED-SD:

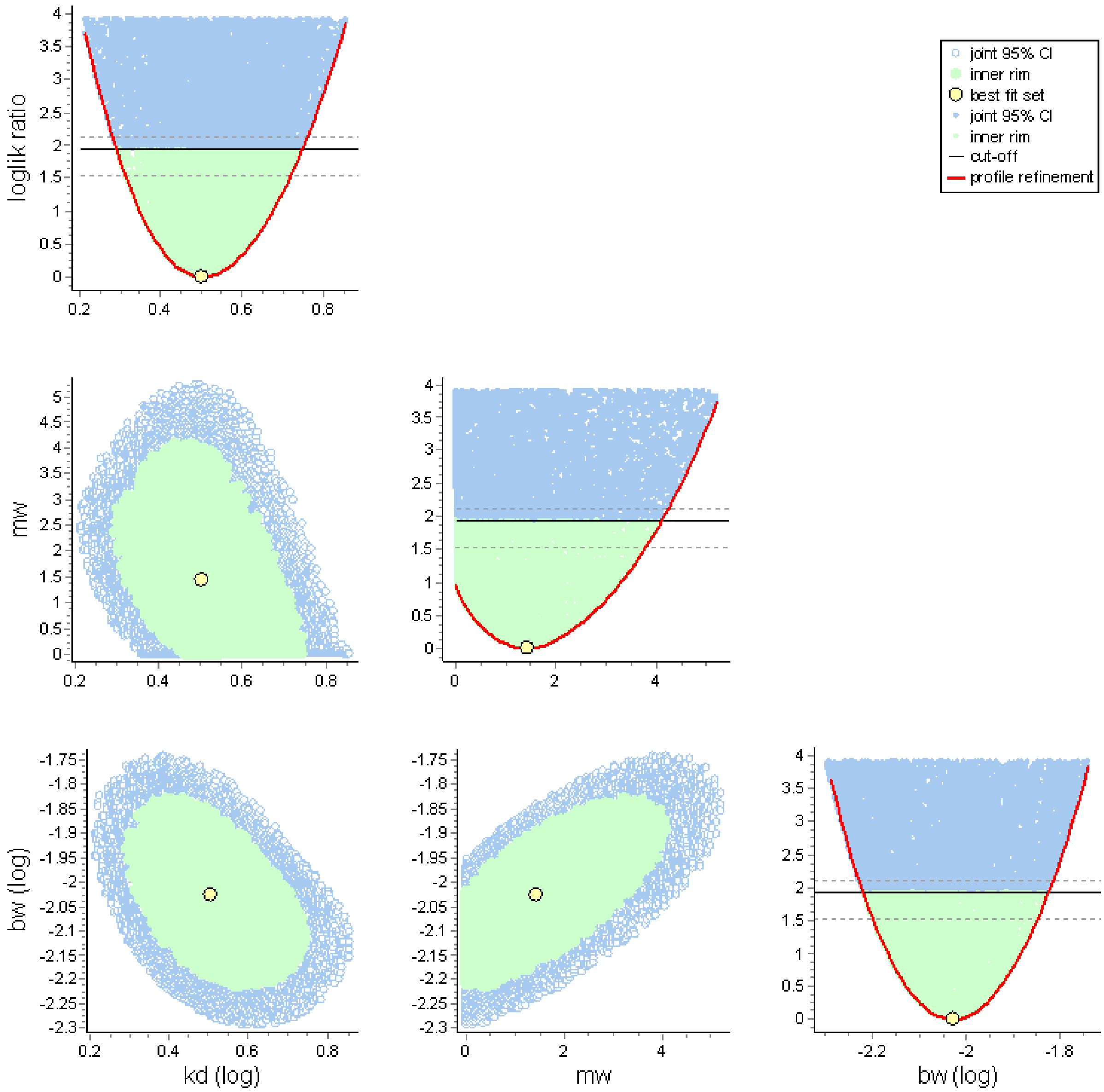

**Exposure, damage and survival plots for the calibration of GUTS-RED-SD:**

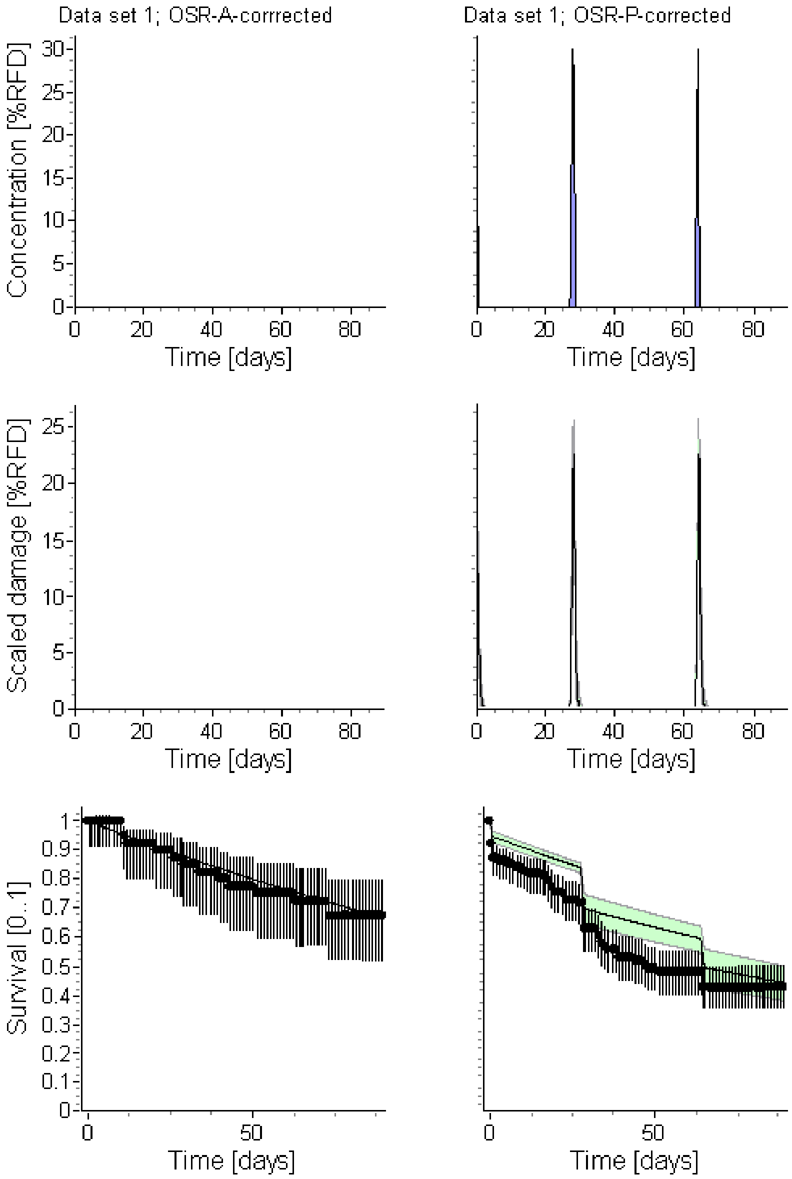

**Observed vs. Predicted survival plot for the calibration of GUTS-RED-SD:**

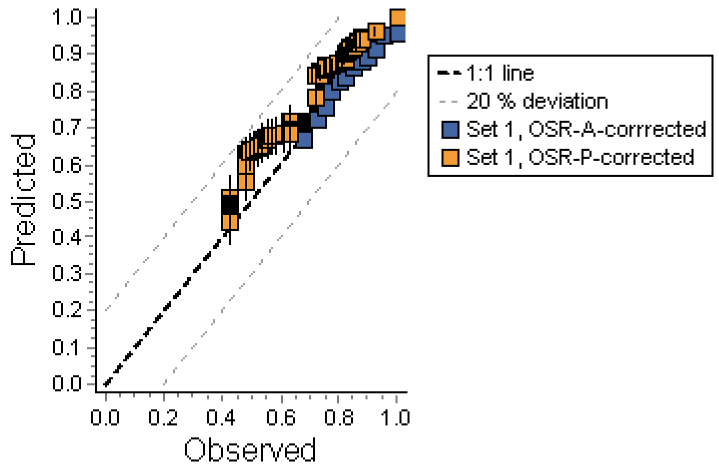


**Observed vs. Predicted deaths plot for the calibration of GUTS-RED-SD:**

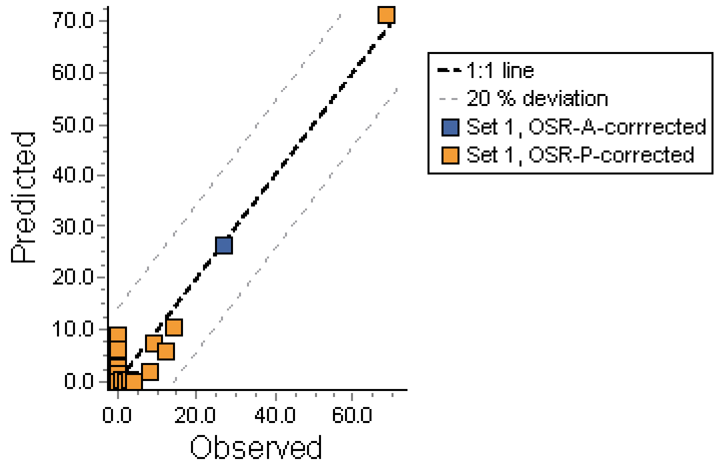


**LCx versus time with confidence intervals (plotted for 16 days, GUTS-RED-SD):**

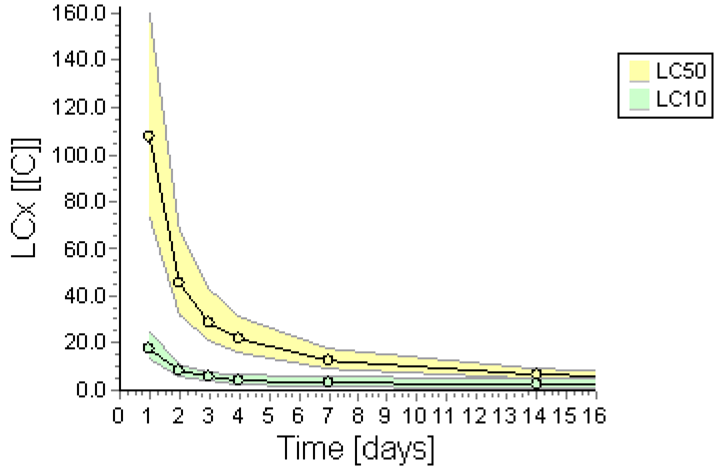

**Fitted parameters for GUTS-RED-IT:**

Best fit parameter values and their 95% CI

kd: 0.024 (0.01916 - 0.0417)

mw: 1.737 (1.194 - 3.014)

Fs: 15.96 (6.665 - 20*)

* edge of 95% parameter CI has run into a boundary

(this may also affect CIs of other parameters)

**Goodness of fit for calibration data (GUTS-RED-IT):**

Model efficiency (NSE, r-square): 0.8097

Normalised root-means-square error (NRMSE): 19.1 %

Minus log-likelihood (MLL): 563.03

AIC: 1132.06

Survival probability prediction error (SPPE) for each treatment:

| Data set | Treatment | Value |
|---|---|---|
| 1 | OSR-A-corrrected | 0.5492 % |
| 1 | OSR-P-corrected | -6.9 % |

**GUTS-RED-IT results table for LCx,t [[C]], with 95% CI:**

| Time [d] | LC50 | LC20 | LC10 |
|---|---|---|---|
| 1 | 73.22 (46.27 - 110.1) | 25.67 (18.54 - 35.66) | 13.9 (9.55 - 19.52) |
| 2 | 37.05 (23.52 - 55.73) | 12.99 (9.397 - 18.05) | 7.036 (4.842 - 9.883) |
| 3 | 24.99 (15.94 - 37.6) | 8.763 (6.351 - 12.18) | 4.746 (3.273 - 6.672) |
| 4 | 18.97 (12.15 - 28.55) | 6.65 (4.829 - 9.245) | 3.602 (2.488 - 5.067) |
| 7 | 11.23 (7.288 - 16.91) | 3.936 (2.874 - 5.476) | 2.132 (1.481 - 3.006) |
| 14 | 6.084 (4.043 - 9.179) | 2.133 (1.568 - 2.973) | 1.155 (0.8106 - 1.636) |
| 21 | 4.386 (2.966 - 6.628) | 1.538 (1.138 - 2.147) | 0.8329 (0.5882 - 1.185) |
| 28 | 3.549 (2.412 - 5.371) | 1.244 (0.9213 - 1.739) | 0.6739 (0.4789 - 0.9646) |
| 42 | 2.734 (1.867 - 4.179) | 0.9586 (0.7104 - 1.345) | 0.5192 (0.371 - 0.7709) |
| 50 | 2.485 (1.7 - 3.84) | 0.8712 (0.6458 - 1.236) | 0.4719 (0.3379 - 0.718) |
| 100 | 1.91 (1.32 - 3.186) | 0.6695 (0.4971 - 1.046) | 0.3627 (0.2568 - 0.6281) |

**Plots for GUTS-RED-IT calibration:**

## Parameter space plot for the calibration of GUTS-RED-IT:

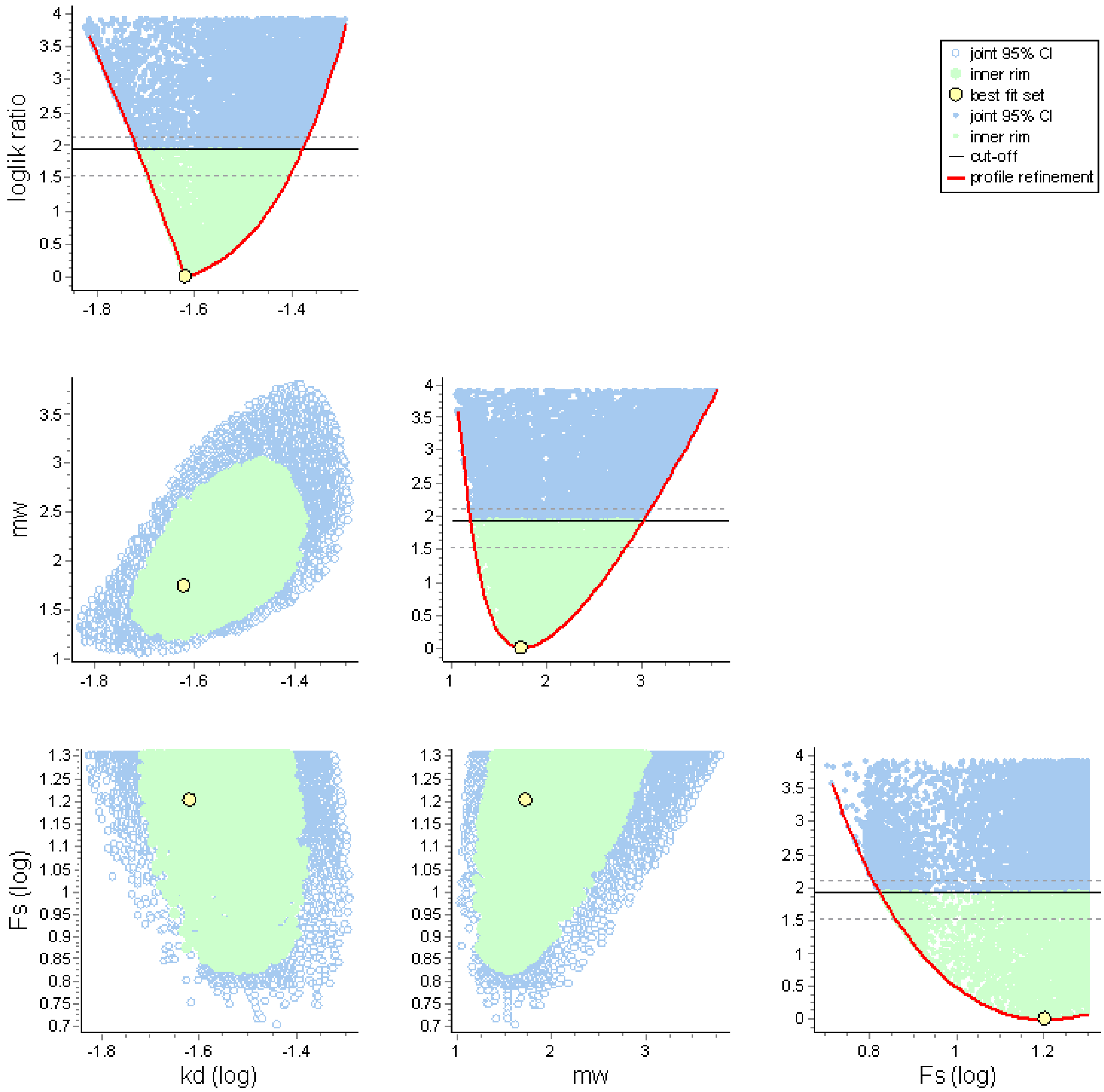

### Exposure, damage and survival plots for the calibration of GUTS-RED-IT:

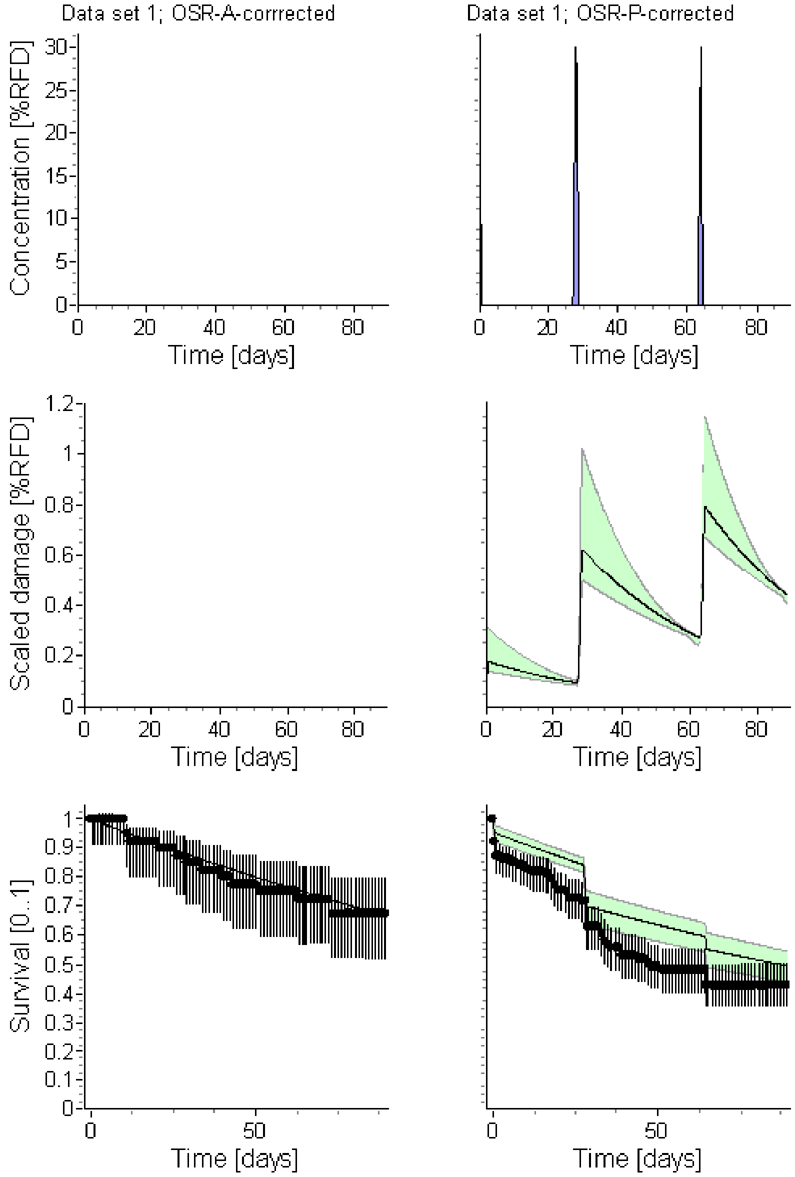

**Observed vs. Predicted survival plot for the calibration of GUTS-RED-IT:**

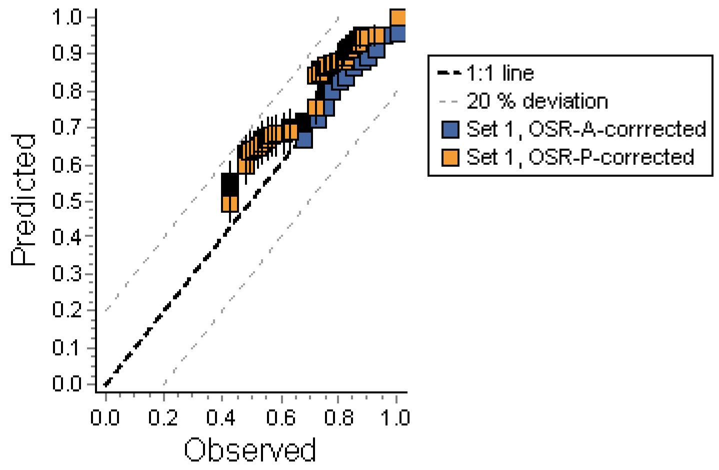


**Observed vs. Predicted deaths plot for the calibration of GUTS-RED-IT:**

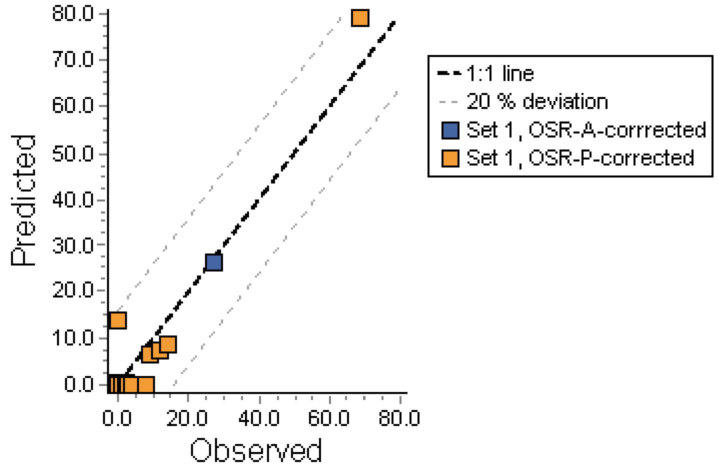


**LCx versus time with confidence intervals (plotted for 16 days, GUTS-RED-IT):**

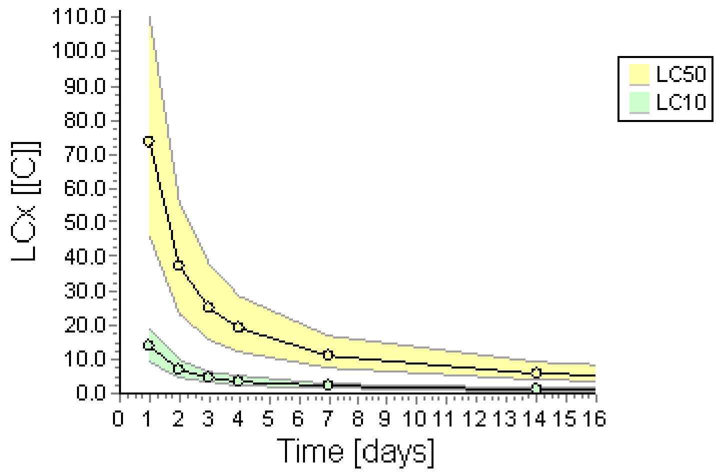

# Validation

No validation performed!

# Predictions

No predictions performed!
